# Immobilization of Glycogen Synthase Kinase-3β Inhibitor on 316L Stainless Steel *via* Polydopamine to Accelerate Endothelialization

**DOI:** 10.3389/fbioe.2021.806151

**Published:** 2021-11-22

**Authors:** Ming Zhang, Xudong Shi, Hai Sun, Donghua Xu, Yang Gao, Xi Wu, Jianqi Zhang, Jichang Zhang

**Affiliations:** ^1^ Cardiology Department, The Second Hospital of Jilin University, Jilin University, Changchun, China; ^2^ Key Laboratory of Polymer Ecomaterials, Changchun Institute of Applied Chemistry, Chinese Academy of Sciences, Changchun, China; ^3^ Jilin Biomedical Polymers Engineering Laboratory, Changchun, China; ^4^ State Key Laboratory of Polymer Physics and Chemistry, Changchun Institute of Applied Chemistry, Chinese Academy of Sciences, Changchun, China

**Keywords:** glycogen synthase kinase-3β inhibitor (GSKi), polydopamine, 316L stainless steel, endothelialization, human coronary artery endothelial cells

## Abstract

The coverage of stents with healthy endothelium is crucial to the success of cardiovascular stent implantation. Immobilizing bioactive molecules on stents is an effective strategy to generate such stents. Glycogen synthase kinase-3β inhibitor (GSKi) is a bioactive molecule that can effectively accelerate vascular endothelialization. In this work, GSKi was covalently conjugated on 316L stainless steel through polydopamine to develop a stable bioactive surface. Fourier transform infrared spectroscopy (FTIR), scanning electron microscopy (SEM) and water contact angle results revealed the successful introduction of GSKi onto 316L stainless steel. The GSKi coating did not obviously affect the hemocompatibility of plates. The adhesion and proliferation of human coronary artery endothelial cells (HCAECs) on stainless steel was significantly promoted by the addition of GSKi. In summary, this work provides a universal and stable strategy of immobilizing GSKi on the stent surface. This method has the potential for widespread application in the modification of vascular stents.

## 1 Introduction

Coronary artery disease (CAD), especially myocardial infarction, is becoming a major cause of death worldwide and is responsible for more than 17.5 million deaths annually (Cui et al., 2018). Drug-eluting stents (DESs) have been successfully used in the treatment of CAD. Despite the success of DESs, a previous study demonstrated that in-stent restenosis (ISR) and late stent thrombosis (LST) represent obstacles to long-term application in the clinic (Otsuka et al., 2012; Liang et al., 2016). It was soon realized that the occurrences of ISR and LST are ultimately due to endothelial injury or the re-endothelialization delay of the stents (Kakinoki et al., 2018). A functionally intact vessel endothelium not only prevents thrombosis but also mediates intimal hyperplasia. As a result, re-endothelialization on the stent effectively prevents ISR and LST (Woods and Marks, 2004). Considerable efforts in the surface modification of stents have been made to improve their ability to rapidly re-endothelialize (Avci-Adali et al., 2010; Qi et al., 2014; Pang et al., 2015). However, the selection of appropriate bioactive molecules and the strategy of immobilizing molecules on stents still need to be investigated.

Glycogen synthase kinase-3β (GSK3β) acts as a nodal point of converging signaling pathways in endothelial cells to adjust vessel growth. It is a pro-apoptotic kinase given that it’s overexpression makes cells sensitive to apoptosis. The inhibitor of GSK3β (GSKi) increases the expression of β-catenin in endothelial cells and promote the expression of vascular endothelial growth factor (VEGF) (Doble and Woodgett, 2003; Skurk et al., 2005; Choi et al., 2007), which is an important regulatory molecule of endothelial cells and plays an important role in vascular development (Ferrara et al., 2003; Eichmann and Simons, 2012). Zhang et al. found that GSKi had a protective effect on endothelial cells by phosphorylating inactivated GSK3β and inhibiting endothelial cell apoptosis (Zhang et al., 2004). Benjamin Hibbert et al. treated endothelial progenitor cells with GSKi to promote the secretion of VEGF and then injected them into immunocompromised mice. They found that GSKi promoted re-endothelialization and reduced neointimal formation after arterial injury (Hibbert et al., 2009). These exciting results indicated that GSKi can be used to accelerate vascular endothelialization after coronary stent implantation. However, the clinical feasibility of transplantation in operant cells *in vitro* may be limited given that the process is labor intensive and impractical.

To date, only a few studies have reported on GSKi-modified stents. Ma et al. coated stents with a mixture of lubricating jelly and active compounds [GSKi and rapamycin (a key DES agent)]. The experimental results revealed that GSKi-coated stents enhance the adhesion of endothelial progenitor cells and efficiently ameliorate the vascular response to stent implantation. Furthermore, GSKi-coated stents can redeem the deleterious endothelial effects of rapamycin-coated stents (Ma et al., 2010). However, this method of coating stents may lead to weak adhesion between the coating and stents and the rapid loss of active compounds caused by simple blending.

In recent years, mussel-inspired polydopamine coatings have attracted researchers’ attention because they have outstanding ability to bind strongly to almost all types of substrates (Lee et al., 2006; Lee et al., 2007a; Lee et al., 2007b; Luo et al., 2013; Zhang et al., 2014; Wu et al., 2016; Zhang et al., 2016; Zhang et al., 2019). Polydopamine coatings can be realized through the simple dip-coating step of the substrates in the aqueous solution of dopamine and provides secondary reactivity for conjugating bioactive molecules. The reaction conditions are mild and easy to perform (Madhurakkat Perikamana et al., 2015; Yang et al., 2015). The bioactive molecules are connected on the material surface by a stable covalent bond between the sulydryl/amine groups of the molecule and the phenolic hydroxyl/quinone groups of polydopamine (Lynge et al., 2011; Qi et al., 2014).

In this study, GSKi was immobilized onto 316L stainless steel plates (316L-SS) by polydopamine coating. The surfaces of the modified 316L-SS were observed by SEM and water contact angle. The hemocompatibility of the modified 316L-SS was studied by blood clotting time tests and platelet adhesion. The viability, adhesion, proliferation and morphology of human coronary artery endothelial cells (HCAECs) on the modified plates were examined using *in vitro* cell experiments.

## 2 Experiments

### 2.1 Preparation of Functionalized Surfaces

The 316L-SS substrates were ultrasonically washed with acetone, ethanol and distilled water for 5 min. The preparation procedure of functionalized surfaces was as follows: 316L-SS was immersed in a 2 mg/L dopamine hydrochloride (Aladdin, United States) solution (dissolved in 10 mM Tris buffer, pH 8.5) for 24 h at room temperature and then rinsed with 10 mM Tris buffer (pH 8.5) thrice to remove the poorly bound polydopamine. The obtained polydopamine-modified stainless steel plates were air-dried and labeled PDAM-SS.

Then, 2 ml GSKi (CHIR-98014, Abmole, United States) solution (2.5 mg/ml DMSO solution) was added to 48 ml distilled water to obtain the supersaturated aqueous solution of GSKi. Then, PDAM-SS was placed into a culture plate and incubated with the supersaturated aqueous solution of GSKi at room temperature for 24 h. Finally, the GSKi-modified samples were rinsed thrice with distilled water and air-dried (labeled GSKi-SS).

### 2.2 Surface Characterization

#### 2.2.1 Fourier Transform Infrared Spectroscopy

Fourier transform infrared spectroscopy (FTIR) (Bruker ALPHA) with the diffuse reflectance mode was used to determine the chemical structure of 316L-SS, PDAM-SS and GSKi-SS.

#### 2.2.2 SEM

The surface morphologies of 316L-SS, PDAM-SS and GSKi-SS were observed using a Gemini 300/VP field emission scanning electron microscope (Zeiss, Germany).

#### 2.2.3 Water Contact Angle

The wettability studies were performed using a contact angle analyzer (DSA10-MK2, Krüss, Germany). The measurement was performed at five different points on each stainless steel plate to obtain the average contact angle.

### 2.3 Hemocompatibility

#### 2.3.1 Activated Partial Thromboplastin Time (APTT)

Platelet-poor plasma (PPP) was obtained by centrifuging fresh human whole blood at 3,000 rpm for 15 min. The samples were incubated with 500 μL PPP in a 24-well plate for 30 min at 37°C. The dotting time of incubated PPP was detected using a coagulometer (ACL TOP 500, Werfen, United States).

#### 2.3.2 Platelet Adhesion

Fresh peripheral blood was obtained from a healthy human volunteer. Platelet-rich plasma (PRP) was obtained by centrifugation of whole blood at 1,500 rpm for 15 min. The samples were placed in a 24-well plate, and 100 μL PRP was added to each sample surface. After incubation at 37°C for 2 h, the PRP on the sample was absorbed and rinsed with PBS thrice. The rinsed samples were then fixed in 2.5% glutaraldehyde for 2 h, rinsed again with PBS thrice, and dehydrated in graded ethanol solution (50, 75, 90, and 100%). Finally, the samples were dried and observed by SEM (Gemini 300/VP field emission scanning electron microscope, Zeiss, Germany).

### 2.4 Cell Experiments

#### 2.4.1 Cell Viability

Complete DMEM was incubated with different samples in a 24-well plate for 24 h to obtain conditioned media, and media without substrate was used as a control. HCAECs were cultured at a density of 3,000 cells per well in a 96-well plate and cultured with normal DMEM complete media overnight. The next day, the media was changed to conditioned media and cultured for 24 h. Then, cell viability was detected using the Cell Counting Kit-8 (CCK-8, Dojindo) assay.

#### 2.4.2 Cell Adhesion and Proliferation

HCAECs were seeded onto different samples at a density of 15,000 cells per well in a 24-well plate. After 2 h of incubation, the cells were fixed in 4% paraformaldehyde for 20 min and blocked with PBS containing 5% BSA for 30 min. Then, 4′,6-diamidino-2-phenylindole (DAPI, 1 μg/ml, Sigma) was used to stain nuclei for 5 min. Then, the adhesion of cells was observed by a confocal laser microscope (LSM 780, Zeiss, Germany). Six images in each channel were obtained for each sample. Image-Pro Plus software was used to calculate the number of cells.

A CCK-8 assay was performed to investigate cell proliferation in different samples. After 24 or 48 h of incubation, 50 μL CCK8 was added to 500 μL culture media and incubated with cells for 1 h at 37°C. One hundred microliters of supernatant was transferred to a 96-well plate, and the optical density was measured with a microplate reader at 450 nm wavelength after the instrument was blanked with PBS solution. Three replicates were prepared for each sample.

#### 2.4.3 Cell Morphology

Cell morphology was determined by DAPI and fluorescein isothiocyanate (FITC)-labeled phalloidin (Sigma) staining. These fluorescent dyes are indicators of the cell nucleus and skeleton. Briefly, HCAECs were incubated on different samples for 2 or 24 h. Then, samples were fixed in 4% paraformaldehyde and blocked with PBS containing 5% BSA for 20 and 30 min, respectively. The cytoskeleton was labeled with 300 nM FITC-labeled phalloidin for 30 min followed by washing with PBS containing 0.1% Tween 20 thrice. DAPI (1 μg/ml) was used to stain nuclei for 5 min. Then, the cells were mounted on slides in glycerol with cover slips and observed with a confocal laser microscope.

### 2.5 Statistical Analysis

All numerical data are presented as the mean ± SD (n ≥ 3). Statistical analysis was performed using one-way ANOVA. If the *p*-value was less than 0.05 (*p < 0.05), the differences were considered statistically significant.

## 3 Results and Discussion

### 3.1 Characterization of Surfaces

**FIGURE 1:**
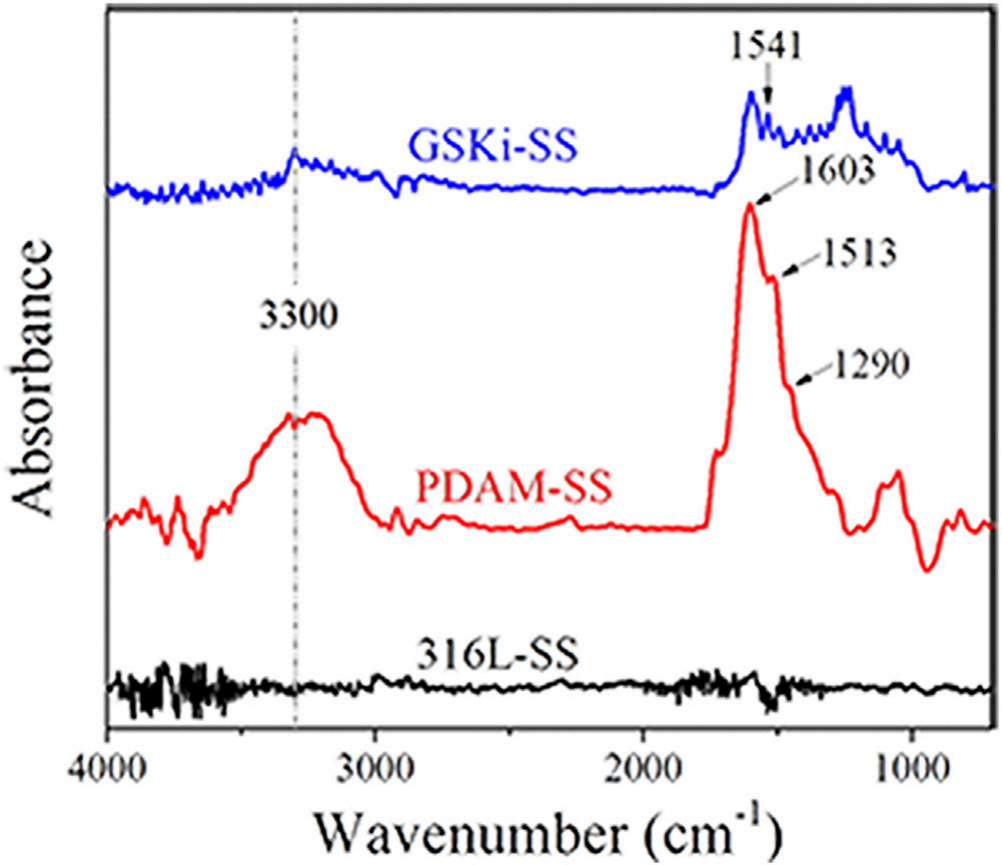
FTIR spectra of 316L-SS, PDAM-SS and GSKi-SS.

The FTIR results in Figure 1 show that 316L-SS has no obvious absorption peak. The characteristic adsorption peaks of PDAM-SS at ∼3,300 cm^−1^ (stretching vibration of phenolic O-H and N-H), 1,603 cm^−1^ (superposition of N-H bending vibration and stretching vibration of aromatic ring), 1,513 cm^−1^ (N-H shearing vibration), and 1,290 cm^−1^ (C-O stretching vibration) proved the existence of polydopamine layer on 316L stainless steel. Compared with PDAM-SS, GSKi-SS exhibits many changes in the FTIR spectrum. The peak at 1,541 cm^−1^ was assigned to amide II (N-H bending vibration), which indicated that GSKi was successfully immobilized on PDAM-SS. The catecholic groups in polydopamine coating offered the active sites to immobilize GSKi molecules.

**FIGURE 2:**
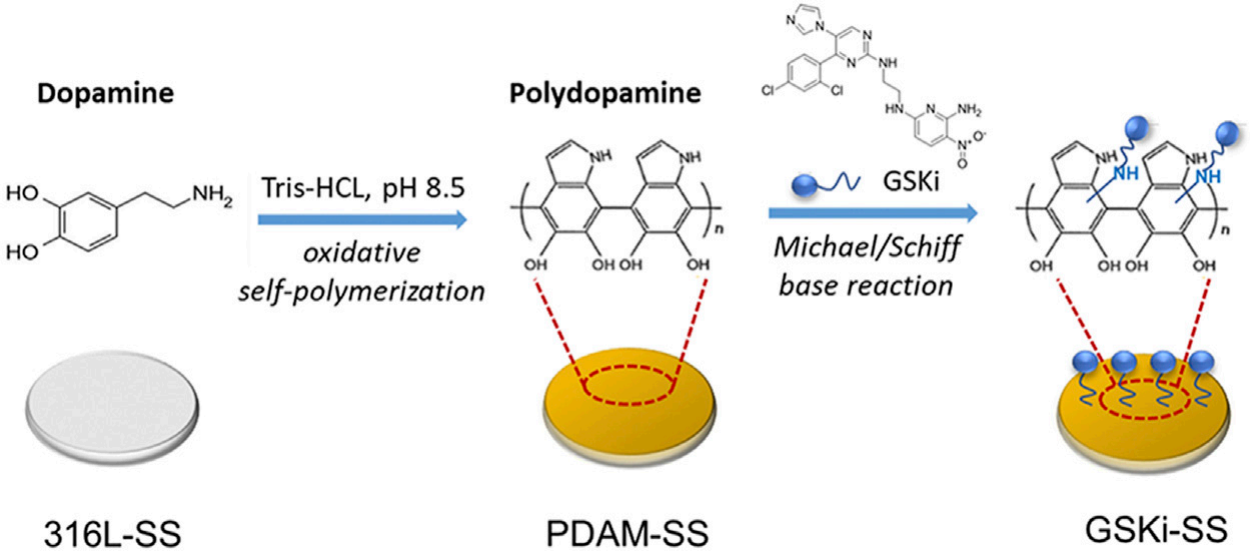
Schematic diagram of the surface modification of 316L-SS.

A schematic diagram of the surface modification procedure of 316L-SS is shown in Figure 2. After 24 h of immersion in dopamine solution, the 31-SS substrates turned light brown in our experiments. The color of PDAM-SS did not change much after immersion in GSKi solution for an additional 24 h. Under weakly alkaline conditions, dopamine self-polymerized and formed a light brown polydopamine coating on the 316L-SS surface. The polydopamine layer is rich in catechol groups. These groups are easily oxidized to phenolic hydroxyl/quinone groups and undergo a Schiff base reaction or a Michael addition reaction with the primary amino group (-NH2) or secondary amino group (−NH) of GSKi (Yang et al., 2014; Madhurakkat Perikamana et al., 2015). Then, GSKi was covalently immobilized on the PDAM-SS surface through polydopamine coating.

**FIGURE 3:**
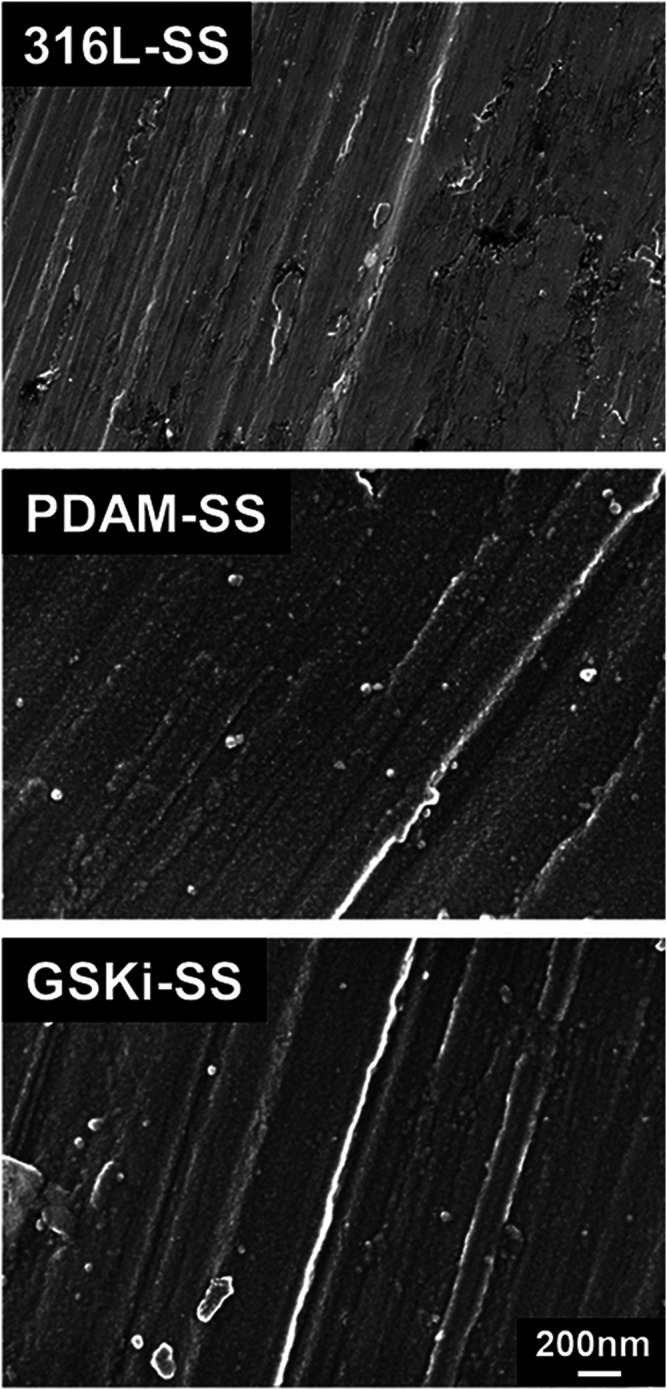
SEM images of 316L-SS, PDAM-SS and GSKi-SS.

The SEM results of 31-SS, PDAM-SS and GSKi-SS are shown in Figure 3. First, the stripes left on the surface of the original 316L-SS were caused by the polishing process. After 24 h of polydopamine coating, the surface morphology of 316L-SS changed significantly. Some nanoscale aggregation was observed on the PDAM-SS surface. However, the surface morphology changed slightly after GSKi was immobilized on the surface of PDAM-SS.

Dopamine is polymerized under weakly alkaline conditions (pH 8.5), and polymerized dopamine can be spontaneously deposited on many types of substrates, such as metals and polymers (Lee et al., 2007a; Yang et al., 2015). After the long-term coating process, polymerized dopamine changed the surface morphology of 316L-SS. It formed a thin adhesive layer and even appeared as nanoaggregates or clumps on the surface of stainless steel plates, which was also reported in other studies (Yang et al., 2012b; Hong et al., 2013; Yang et al., 2015).

The surface hydrophobicity was evaluated by measuring the water contact angle of each sample. The results of 316L-SS, PDAM-SS and GSKi-SS are 53.9 ± 7.4°, 6.4 ± 1.1° and 40.7 ± 2.3°, respectively. The addition of polydopamine obviously improved the surface hydrophilicity of stainless steel plates. GSKi-SS is more hydrophobic than PDAM-SS.

The large amount of hydrophilic functional groups, such as phenolic hydroxyl groups and amino groups of the polydopamine coating, are potentially responsible for the improved hydrophilicity of PDAM-SS (Yang et al., 2010; Ding et al., 2014). The increased hydrophobicity of GSKi-SS may be due to the connection of GSKi with the phenolic hydroxyl group of the polydopamine coating, which resulted in a significant reduction of the hydrophilic group. The change in contact angle of the three samples indirectly demonstrated the successful coating of polydopamine and GSKi on the 316L-SS surface.

### 3.2 Hemocompatibility

**FIGURE 4:**
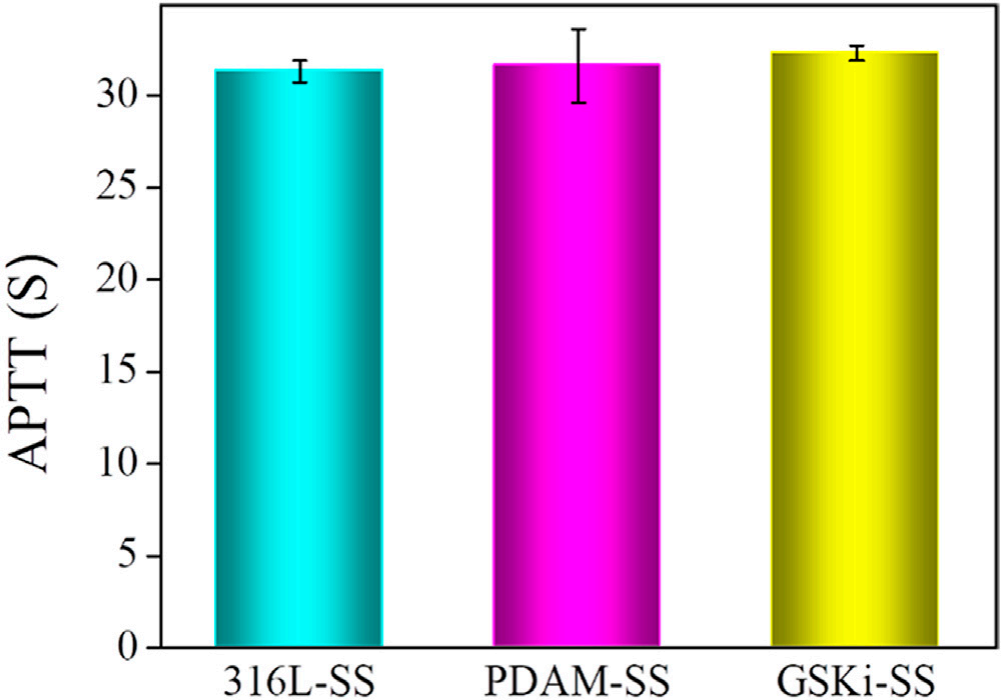
APTT evaluation of 316L-SS, PDAM-SS and GSKi-SS (mean ± SD, *N* = 3).

For cardiovascular implant devices, improperly modified materials can trigger thrombus cascade reactions, which lead to implantation failure. APTT is a simple and highly reliable assay for the evaluation of the anticoagulant capacity of material surfaces (Lu et al., 2012). When stents with poor blood compatibility are implanted, the coagulation factor will bind to the stent surface to initiate the endogenous coagulation reaction, and the clotting time will be reduced during APTT detection (Basmadjian et al., 1997). The APTT results of 316L-SS, PDAM-SS, and GSKi-SS are shown in Figure 4. Compared with 316L-SS (31.33 ± 0.55 s), the values of PDAM-SS (31.63 ± 1.95 s) and GSKi-SS (32.3 ± 0.44 s) were not drastically altered. No significant difference was noted among the three groups (*p* > 0.05), indicating that these groups have similar anticoagulant properties. Therefore, the polydopamine and GSKi coating on 316L-SS did not obviously activate the endogenous coagulation system.

**FIGURE 5:**
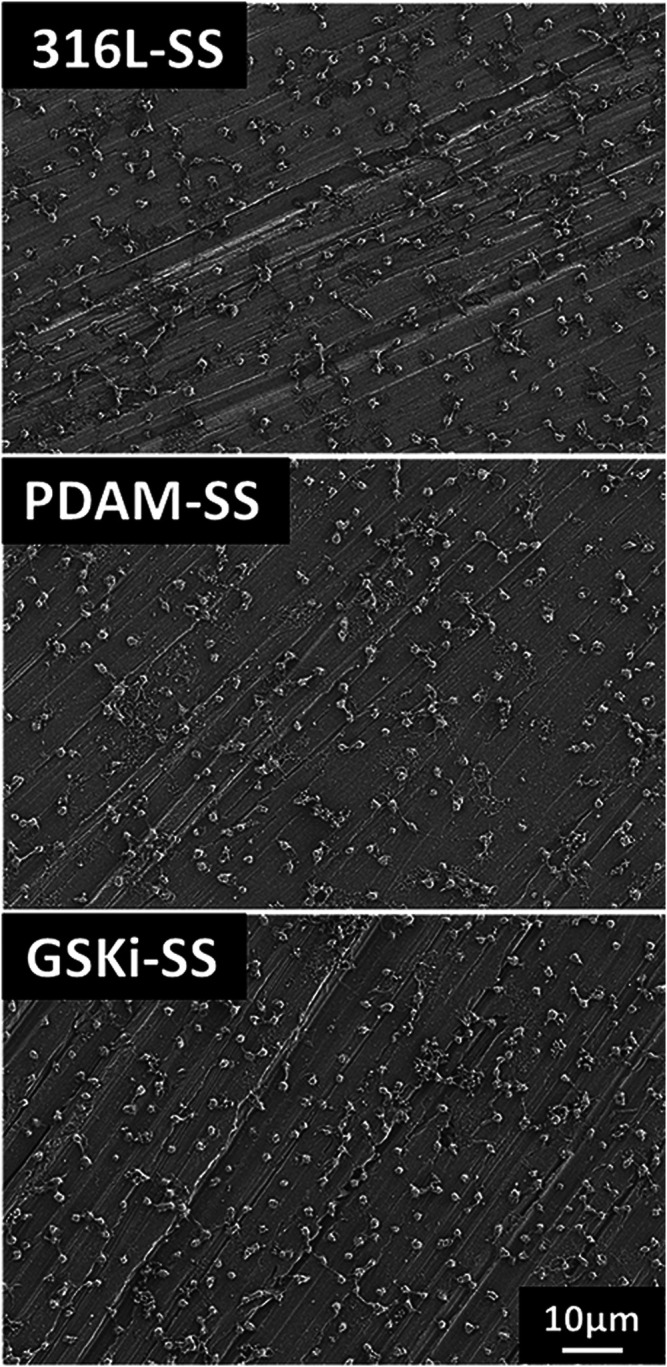
Platelet adhesion on 316L-SS, PDAM-SS and GSKi-SS after contact with PRP for 2 h.

As a key step of thrombosis, platelet adhesion has been widely used to characterize the thrombogenic ability of materials. The adhesion morphology of platelets on materials can be divided into five grades: discoid, dendritic, partially spreading, spreading and aggregation (Goodman et al., 1989). To study the thrombogenic properties of PDAM- and GSKi-modified 316L stainless steel, platelets adhered to these sample surfaces were observed by SEM (as shown in Figure 5). The amount and morphology of platelets were similar in the three groups. All platelets showed discoid or partial dendritic morphology. This SEM result indicated that 316L-SS, PDAM-SS and GSKi-SS had similar anticoagulant properties, which was consistent with the results of the APTT test. Therefore, the polydopamine and GSKi coating on 316L-SS did not obviously activate the endogenous coagulation system.

### 3.3 Cellular Responses

**FIGURE 6:**
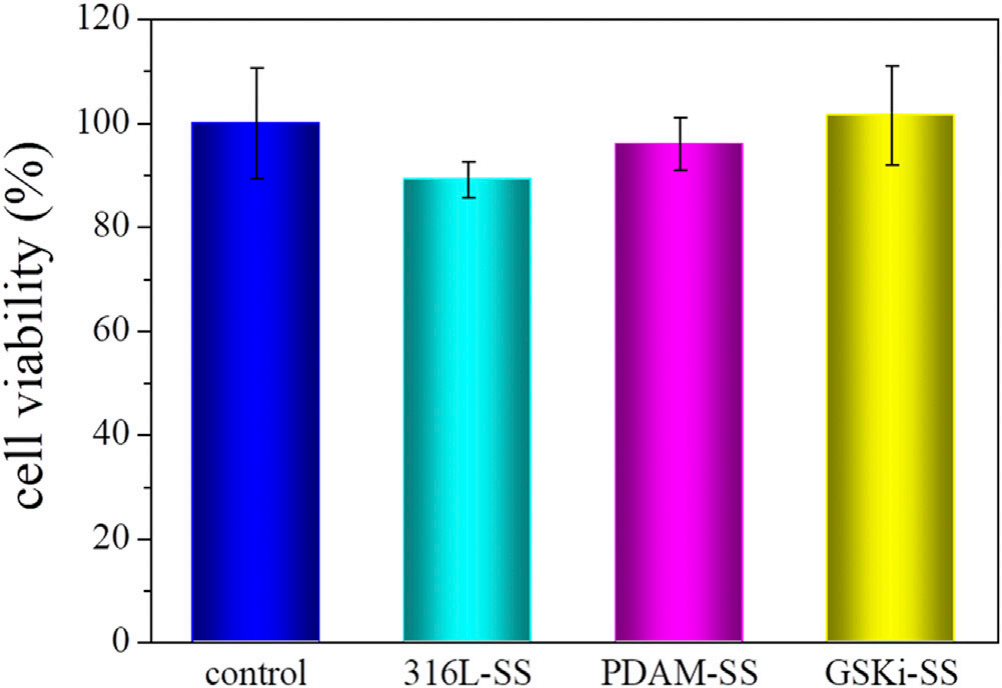
HCAEC viability after incubation with 316L-SS, PDAM-SS and GSKi-SS for 24 h.

To explore the neovascularization ability of modified stainless steel plates, it is necessary to study the growth behaviors of HCAECs in the three groups. Figure 6 shows the cell cytotoxicity of 316L-SS, PDAM-SS and GSKi-SS after 24 h of cell culture. The tissue culture plate served as a control. The cell viability of each sample was not significantly different from that of the control group and was greater than 85%. This finding indicated that 316L-SS, PDAM-SS and GSKi-SS had good biocompatibility. The polydopamine and GSKi coating of 316L-SS did not lead to obvious cell cytotoxicity.

**FIGURE 7:**
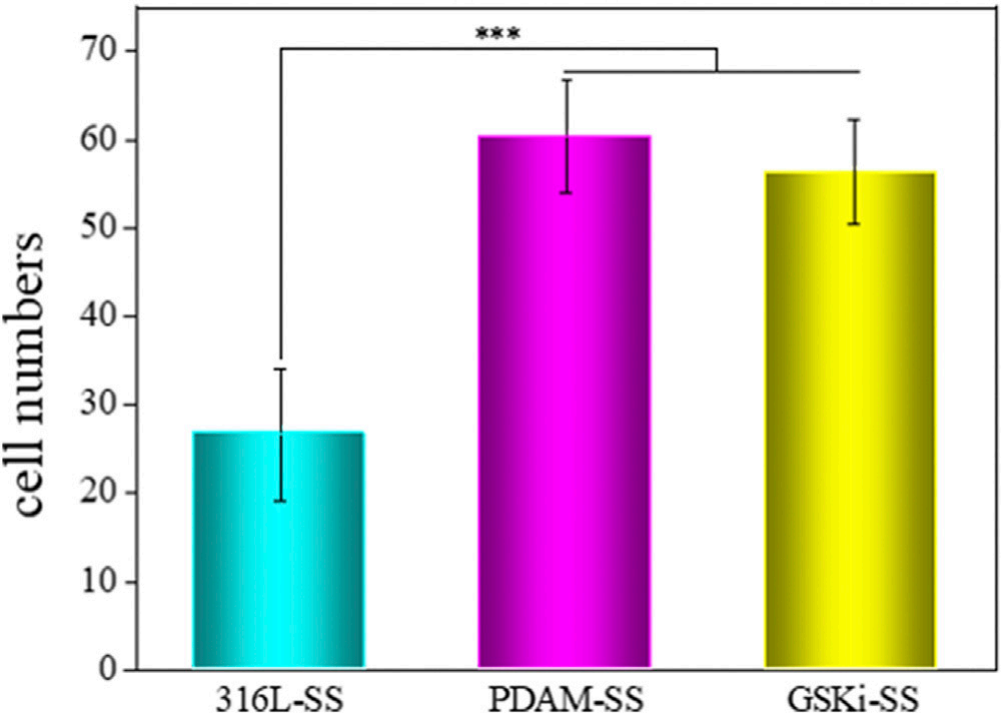
Cell adhesion numbers on various samples after 2 h of incubation (mean ± SD, ***p < 0.001).

The adhesion and proliferation of endothelial cells on stent surfaces is necessary for the re-endothelialization of stents. Figure 7 shows the number of cells attached to different stainless steel samples after 2 h of incubation. The cell adhesion numbers for PDAM-SS and GSKi-SS were significantly greater than that of 316L-SS. No significant difference was noted between PDAM-SS and GSKi-SS. The surface properties of materials, such as topography, wettability and functional groups, play important roles in regulating cell behaviors (Flemming et al., 1999; Alves et al., 2010; Yuan et al., 2018; Amani et al., 2019; Shi et al., 2019). In this study, nanopolydopamine dots that appeared on the surface of PDAM-SS and GSKi-SS were thought to contribute to improving the adhesion of HCAECs. The strong protein adsorption capacity of the polydopamine coating also promoted the adhesion of cells on PDAM-SS (Ku et al., 2010; Yang et al., 2012b). Ma et al. reported that GSKi increases the attachment of endothelial progenitor cells to GSKi-coated stents (Ma et al., 2010). In our experiments, the HCAEC attachment efficiency of GSKi-SS was similar to that of PDAM-SS when cells were cultured for 2 h. The cell adhesion results indicated that PDAM-SS and GSKi-SS provided a more suitable microenvironment for the adhesion of HCAECs than 316L-SS.

**FIGURE 8:**
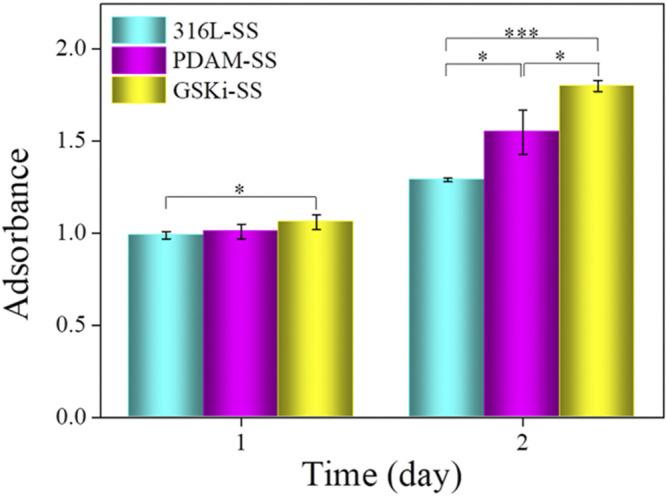
Proliferation of HCAECs on various samples under serum condition (mean ± SD, *p < 0.05, ***p < 0.001).

The cell proliferation results of different samples are shown in Figure 8. Compared with 316L-SS, PDAM-SS did not obviously promote HCAEC proliferation until 48 h of culture. For the GSKi-SS group, cell proliferation increased after incubation for 24 h and was further significantly enhanced after 48 h. These results showed that both polydopamine and GSKi coating on stainless steel plates can promote HCAEC proliferation. GSKi more effectively improves cell proliferation than PDAM. GSKi protects endothelial cells by phosphorylating inactivated GSK3β, resulting in the accumulation of β-catenin in the endothelial nucleus, activating the Wnt signaling pathway and inhibiting endothelial cell apoptosis (Zhang et al., 2004). Choi et al. reported that GSKi stimulates VEGF expression in endothelial cells (Choi et al., 2004). *In vitro* studies we found that VEGF-immobilized surfaces accelerate endothelialization by increasing the adhesion, proliferation and migration of endothelial cells (Shin et al., 2012). Therefore, GSKi-SS yields better cell proliferation results than 316L-SS and PDAM-SS.

**FIGURE 9:**
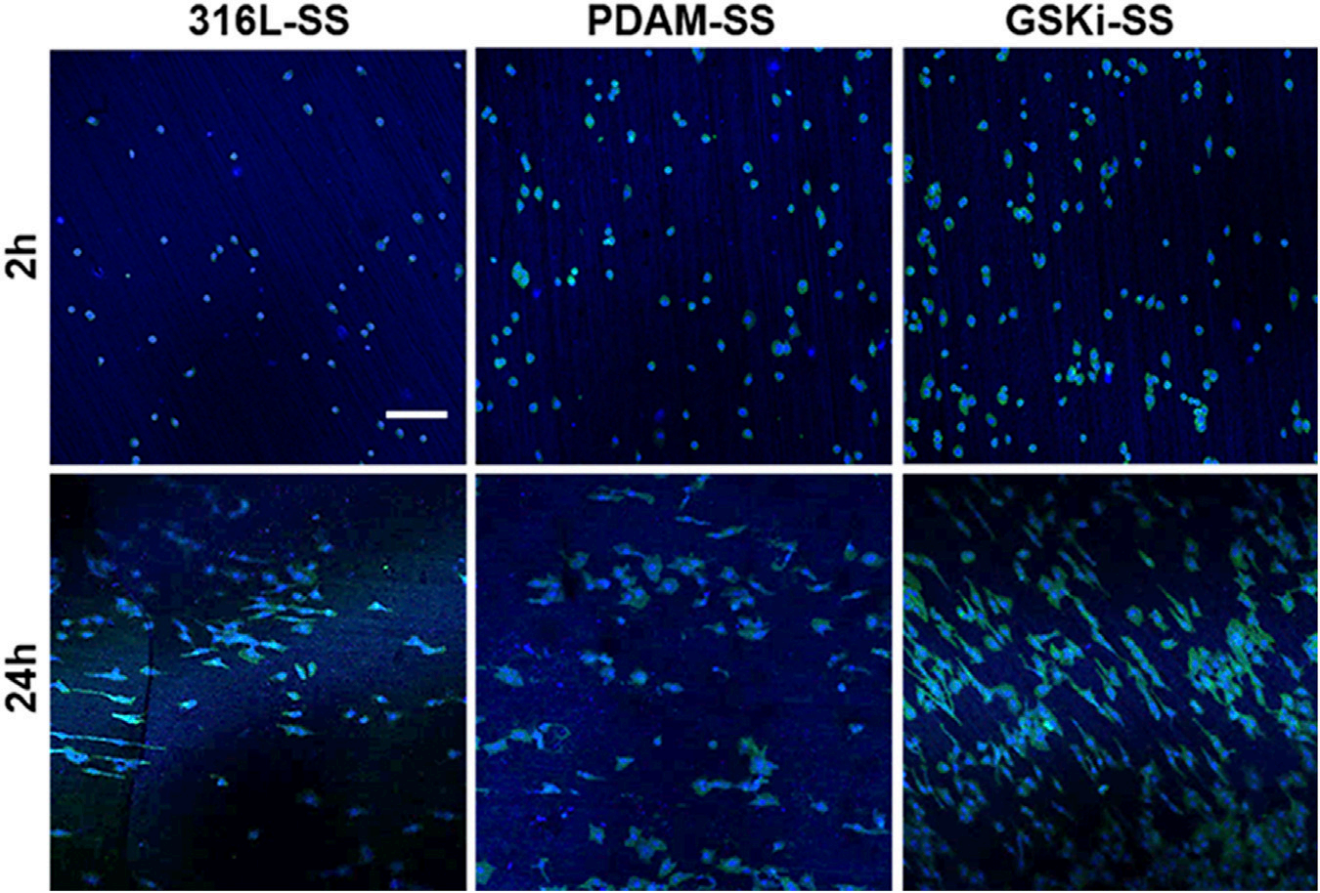
Fluorescence images of HCAECs cultured on different samples for 2 and 24 h; the scale bar is 200 μm.

The morphology of HCAECs on different samples is shown in Figure 9. After culturing for 2 h, only a few cells adhered to 316L-SS. The number of adherent cells on PDAM-SS and GSKi-SS was significantly increased. Cells on PDAM-SS and GSKi-SS began to spread and showed similar morphological features. After 24 h of culture, the cell numbers obviously increased, and the cells completely spread on all three samples. GSKi-SS showed a significant increase in cell coverage and proliferation compared with PDAM-SS and 316L-SS.

The nano and microscale architecture of material surfaces can modulate endothelial cell structure and function (Leclech et al., 2020). Cells on along the microgrooves on the stainless steel surface in the GSKi-SS samples exhibited a more elongated, unidirectional and polarized morphology than those in the 316L-SS and PDAM-SS groups. These slender HCAECs were thought to not only have stronger proliferative capacity and extracellular matrix secretion but also have a faster migration rate than fully spread cells (Yang et al., 2012a). This fluorescence result was consistent with the cell adhesion and proliferation results shown in Figure 8.

## 4 Conclusion

In this study, GSKi was successfully introduced on the surface of 316L stainless steel through polydopamine to form a stable bioactive coating. Blood compatibility tests indicated that the addition of GSKi did not obviously affect the hemocompatibility of 316L-SS. Cell experiments revealed that the GSKi coating significantly promoted the adhesion and proliferation of HCAECs on stainless steel surfaces. Compared with 316L-SS and PDAM-SS, cells on GSKi-SS exhibited a more elongated, unidirectional and polarized morphology along the microgrooves on the stainless steel surface. These results suggested that this surface modification strategy of stable GSKi coating has potential application in the modification of cardiovascular stents.

## Data Availability

The original contributions presented in the study are included in the article/Supplementary Material, further inquiries can be directed to the corresponding author.
